# Impact of Konjac Glucomannan with Different Molecular Weight on Retrogradation Properties of Pea Starch

**DOI:** 10.3390/gels8100651

**Published:** 2022-10-13

**Authors:** Shishuai Wang, Shuo Chen, Lidong Ding, Ying Zhang, Jiaxin He, Bin Li

**Affiliations:** 1 College of Food Science and Technology, Wuhan Business University, Wuhan 430056, China; 2 College of Food Science and Technology, Huazhong Agricultural University, Wuhan 430070, China

**Keywords:** konjac glucomannan, pea starch, retrogradation, water holding capacity, crystallinity

## Abstract

The impact of konjac glucomannan (KGM) with different molecular weight (Mw) on the retrogradation properties of pea starch, such as color, viscoelasticity, gel strength, water holding capacity (WHC), moisture distribution and crystallinity, was investigated. At the same time as the Mw of KGM decreased, the lightness, elastic modulus, gel strength, water freedom and crystallinity of pea starch showed an increasing trend, whereas the viscosity modulus and WHC showed a decreasing trend. At one day of storage, compared with single pea starch, KGM with low Mw made gel strength increase from 40 g to 45 g, WHC decrease from 82% to 65% and crystallinity increase from 21.3% to 24.0%. Therefore, KGM with low Mw could promote retrogradation of pea starch in the short-term. At 7 days or even 14 days of storage, KGM with medium-high Mw had smaller indices than those of pure pea starch, including the lightness, storage modulus, gel strength, water freedom and crystallinity. This indicated that KGM with medium-high Mw could inhibit the long-term retrogradation of starch. The larger the Mw of KGM, the more noticeable the inhibition effect.

## 1. Introduction

Pea starch (PS) is widely available and inexpensive. Compared with cereal starch and potato starch, its amylose content is higher, accounting for about 30–65% of the total starch [1]. Pea starch has the advantages of high gel strength and easy regeneration, and is suitable for the processing of vermicelli and jelly. Undeniably, these features make it an excellent material for the preparation of gelatinous food. However, such fresh and wet food is prone to harden, dry shrinkage and dehydration during transportation and storage, resulting in a decline of edible quality, which is also caused by the retrogradation of starch [2].

The retrogradation of starch is defined as the recombination of starch chains (amylose and amylopectin) in the gelatinized paste, giving rise to the formation of a more ordered structure [3]. Regulating the retrogradation of starch has been a hotspot in the food industry. It has been reported that non-starch polysaccharides can delay or inhibit starch aging to some extent, improve the sensory quality of food products, and maintain food quality during processing and storage [4,5].

Konjac glucomannan (KGM) is a kind of neutral polysaccharide extracted from the tubers of konjac. It is mainly composed of D-glucose and D-mannose, which are linked by β-(1,4) glycosidic bonds in a molar ratio of 1:1.6 [6]. Due to its high molecular weight and viscosity, KGM has been widely used as a thickener, gelling agent and edible coating [7,8,9,10]. Currently, some of the literature has reported that KGM could inhibit the retrogradation of starch. For example, Lin pointed out that as KGM concentration increased from 0 to 0.30%, mung bean resistant starch dropped from 71.89% to 57.71% [11]. Guo found that the addition of KGM noticeably caused the decline of the pasting temperature of wheat starch and the increase of the pasting viscosity, breakdown value and setback value, accompanied by the inhibition of the syneresis and retrogradation of the starch gel [12]. Schwartz discovered that 1% KGM in a starch suspension contributed to a slight reduction in the content of available water, for it seemed to disturb the gelatinization and the retrogradation of broad bean starch and potato starch [13]. However, there were other reports that KGM could promote starch retrogradation. For example, Ning found that as KGM content increased from 3% to 12%, the crystallinity of debranched corn starch increased from 14.5% to 18.75%, and the crystallization type of remained the same. KGM tend to aggregate with each other, forcing amylose into smaller spaces, which was conducive to the recrystallization of amylose during rest [14]. Song discovered that the interaction between indica rice starch and KGM promoted the formation of retrograded starch as KGM concentration increased from 0% to 0.2%. However, when the concentration of KGM was greater than 0.3%, the amount of retrograded starch did not change significantly [15].

Moreover, the molecular weight (Mw) of the polysaccharide also has an influence on the aging properties of starch. For the same polysaccharide, the larger the Mw, the more effective it was at restraining starch aging, such as wheat bran arabinoxylan [16], water-extractable arabinoxylan [17], corn fiber gum [18], dextran [19] and pectin [20]. By contrast, the smaller the Mw of the same polysaccharide, the more significant it was at inhibiting starch aging, including water-extractable arabinoxylan [17], inulin [21] and branched limit dextrins [22]. It is supposed that the Mw of a polysaccharide changes its spatial conformation, viscosity, water holding capacity and interactions with starch molecules, thus affecting the aging process of starch.

However, there are few reports concerning the influence of KGM with various Mw on the retrogradation properties of pea starch. Therefore, the aim of this work was to explore the impact of KGM with different Mw at the fixed mixing ratio on the retrogradation properties of pea starch using a colorimeter, a dynamic rheometer, a textural analyzer, water holding capacity (WHC), low-field nuclear magnetic resonance (LF-NMR) and an X-ray diffractometer (XRD).

## 2. Results and Discussion

### 2.1. Lightness (L)

**Figure 1:**
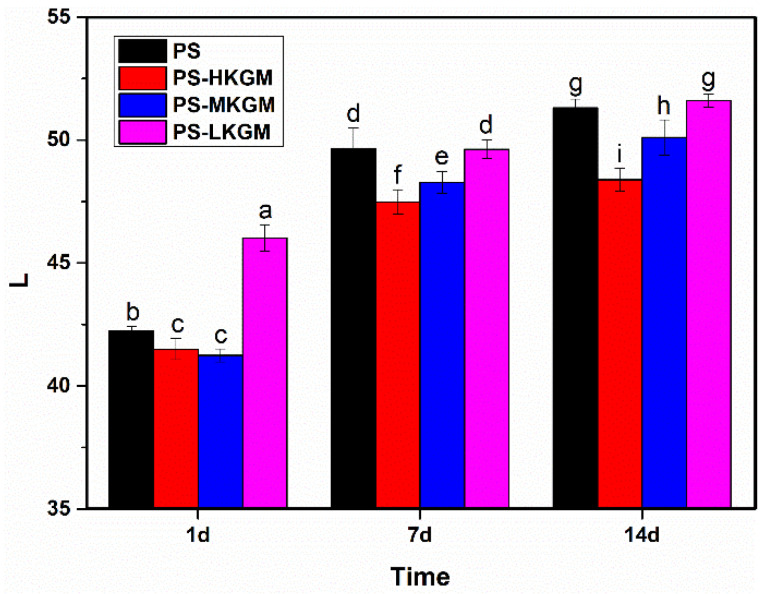
L values of PS—KGM mixtures at different storage times (HKGM, MKGM and LKGM represent KGM with high, medium and low molecular weight, respectively. PS represents pea starch. Different letters indicate significant differences at the *p* < 0.05 level).

In order to explore the effects of the Mw of a polysaccharide on the retrogradation properties of starch, three different Mw of KGM were prepared using hydrothermal degradation. Their Mw were 1.5 × 10^6^ Da, 1.0 × 10^6^ Da and 3.9 × 10^5^ Da, which were labeled as HKGM, MKGM and LKGM, respectively. Figure 1 shows the effects of KGM with various Mw on the L values of pea starch at different storage times. Molecular rearrangement takes place in the process of starch retrogradation, accompanied by the reflection and refraction of light, which results in an increase of turbidity corresponding to the increase of the L value [23]. From the figure it was observed that the L values of all samples increased with time in the presence or absence of KGM, which indicated that the retrogradation of starch was occurring continuously.

At one day of storage, the L value of pea starch blended with LKGM was significantly higher than the others. However, as the storage time was extended to 7 days or even 14 days, the L values of PS-LKGM were almost equal to those of pure pea starch. It was speculated that LKGM could promote regeneration of pea starch in the short-term but had no effect on starch regeneration in the long-term.

For the other two Mw of KGM, the L values of HKGM and MKGM were lower than that of single pea starch throughout the storage period. Especially in the late stages, it was evident that the larger the Mw of KGM, the smaller the L value. Accordingly, it was supposed that KGM with medium and high Mw could inhibit the long-term retrogradation of starch.

### 2.2. Dynamic Viscoelastic Properties

**Figure 2:**
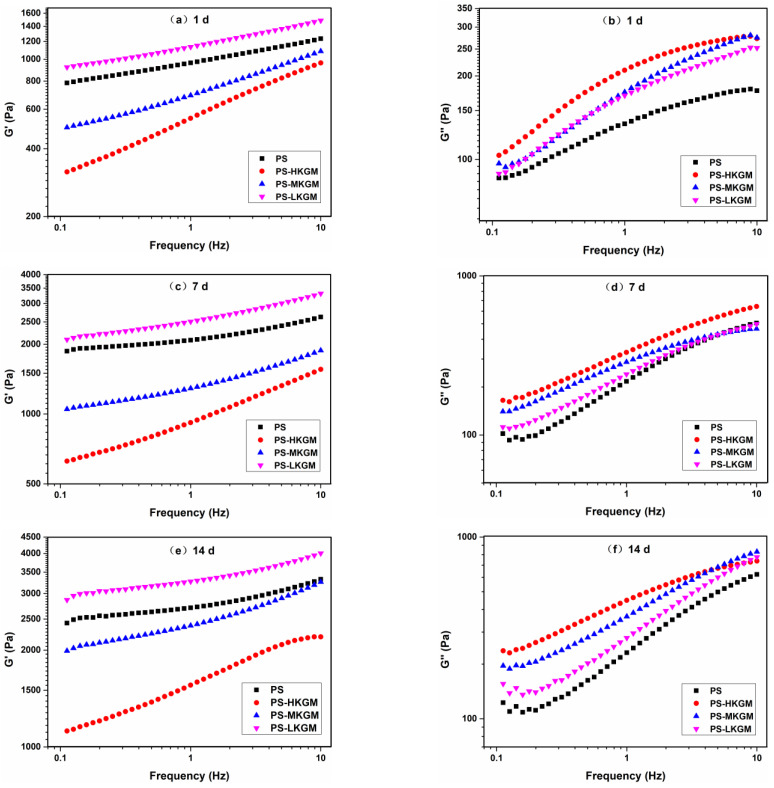
Frequency sweeping curves of PS-KGM mixtures at different storage times are as follows: (**a**) storage modulus at 1st day; (**b**) loss modulus at 1st day; (**c**) storage modulus at 7th day; (**d**) loss modulus at 7th day; (**e**) storage modulus at 14th day; (**f**) loss modulus at 14th day (HKGM, MKGM and LKGM represent KGM with high, medium and low molecular weight, respectively. PS represents pea starch).

The influence of frequency on the storage modulus G′ and the loss modulus G″ of PS- KGM mixtures at different times is depicted in Figure 2. Within the frequency range of 0.1–10 Hz, G′ exceeded G″ without any crossover. Moreover, the magnitude of G′ and G″ from all samples increased with a small frequency dependence, which displayed a typical weak gel-like viscoelastic behavior [24,25]. The G′ of each sample increased with time, which revealed that starch molecules were constantly rearranging and assembling, which caused the increasing elasticity of gel structures.

At the same time, the order of G′ from all samples was PS-LKGM > PS > PS-MKGM > PS-HKGM. Similar phenomena were reported that at the same angular frequency starch gels with HWBAX (wheat bran arabinoxylan with a larger Mw and branching degree) revealed the smaller G′ compared to starch paste containing the same amount of LWBAX (wheat bran arabinoxylan lower Mw and branching degree) [16]. Moreover, the order of G″ from blends under the same time condition was as follows: PS-HKGM > PS-MKGM > PS-LKGM > PS. The larger the Mw of KGM, the greater the G″ of the sample. Overall, compared with pea starch alone, high Mw of KGM decreased the G′ and increased the G″ of the mixtures. Conversely, low Mw of KGM increased the G′ of the mixtures and had a small contribution to the G″ of the mixtures. The reason for this might be the thermodynamic incompatibility between KGM and pea starch, resulting in microphase separation. On the one hand, the microphase regions formed by KGM enrichment were distributed in starch paste, leading to defects in the three-dimensional network structure of the starch molecules and reducing the density of effective cross-linking in the blends, which was manifested as a decrease in G′ [26]. On the other hand, phase separation could increase the concentration of starch molecules so that starch molecules were cross-linked at a higher density, which was shown by the increase in G′ [27]. Different Mw of KGM could cause the two opposite effects of phase separation. The role of HKGM was mainly reflected in the former, whereas the role of LKGM was mainly reflected in the latter.

### 2.3. Gel Strength

**Figure 3:**
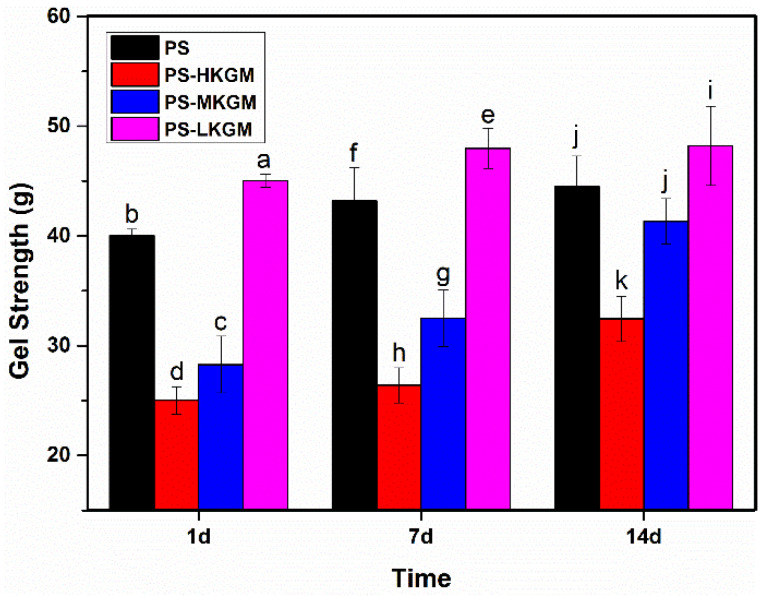
Gel Strength of PS—KGM mixtures at different storage times (HKGM, MKGM and LKGM represent KGM with high, medium and low molecular weight, respectively. PS represents pea starch. Different letters indicate significant differences at the *p* < 0.05 level).

There is a good correlation between the hardness of food and the aging of starch. As time persists, the hardness of starch-based food becomes greater, and the sensory quality of food decreases, which is attributed to the aging of starch. Therefore, the gel strength was measured to reflect the hardness of gel and starch aging [28]. The effect of KGM with various Mw on the gel strength of pea starch at different storage times is shown in Figure 3. The gel strength of each sample showed an upward trend over time, which again confirmed the occurrence of starch aging. The larger the Mw of KGM, the smaller the gel strength of starch paste. When the storage period was just one day, the gel strength of single pea starch reached 40 g. After the addition of HKGM, the gel strength of pea starch decreased significantly to 25 g. However, LKGM caused the gel strength increase to 45 g. The longer molecular chain of KGM hindered the rearrangement and aggregation of starch molecules, weakened the intermolecular interaction of starch and led to the decrease of gel strength [29]. This phenomenon was consistent with the rheological results.

### 2.4. Water Holding Capacity (WHC)

**Figure 4:**
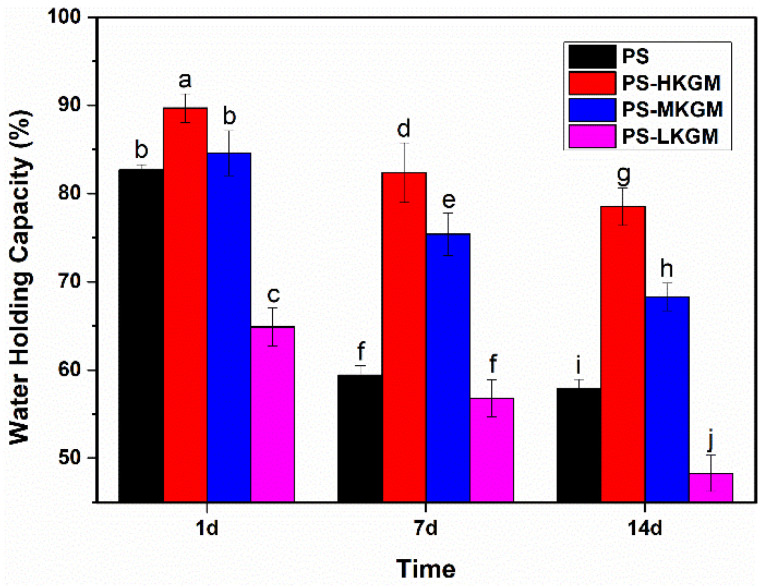
Water holding capacity of PS—KGM mixtures at different storage times (HKGM, MKGM and LKGM represented KGM with high, medium and low molecular weight, respectively. PS represents pea starch. Different letters indicate significant differences at the *p* < 0.05 level).

Starch retrogradation is accompanied by dehydration, resulting in the degradation of food sensory quality. Therefore, WHC can be adopted to reflect the regeneration degree of starch indirectly. If the WHC of the sample increased, the retrogradation degree of starch declined. The effect of various Mw of KGM on the WHC of pea starch with time is presented in Figure 4. Compared with single pea starch, the WHC of pea starch mixed with HKGM or MKGM was significantly greater at any time; moreover, the larger the Mw of KGM, the stronger the WHC of the samples. When the storage time was prolonged from 1 day to 14 days, the WHC of single pea starch dropped from 82% to less than 60%, the WHC of PS-HKGM dropped from 90% to 80%, and the WHC of PS-MKGM dropped from 85% to 68%. Although the WHC of all samples decreased with time, KGM with medium or high Mw to pea starch helped to maintain the network structure of starch gel and reduce the dehydration of the samples. The larger the Mw of KGM, the more remarkable the effects of suppressing dehydration [30].

In addition, it was worth noting that the WHC of PS-LKGM was lower than that of pure pea starch during the entire storage period. At one day of storage, LKGM caused the WHC of pea starch decline to 65%. KGM with small Mw not only was not conducive to the water retention of the samples but also accelerated water loss and promoted regeneration to a certain extent.

### 2.5. Moisture Distribution

Low-field nuclear magnetic resonance (LF-NMR) is a fast and non-destructive detection method, which is used to analyze the motion of water molecule in food systems. The spin–spin relaxation time (T_2_) can represent the different molecular mobility as well as the combination degree between the sample and moisture. A lower T_2_ indicates the closer binding of water molecules in the samples [31]. Table 1 shows the T_2_ values of PS-KGM mixtures at different storage times. The T_2_ value of each sample decreased over time. It was known that the retrogradation of starch required the participation of water. During the rearrangement of starch, a part of the water became increasingly stable in the system, which caused the decline of T_2_ values [32]. Moreover, we found that the addition of KGM changed the distribution and freedom of water in pea starch paste. In the single pea starch, a noticeable peak signal appeared at the range of 43.29~72.38 ms. When various Mw of KGM were added to starch paste, two distinct peak signals appeared, which were located around 10 ms and 200 ms, respectively. A similar phenomenon has been reported in blends of pullulan-rice starch [33], KGM-corn starch [27], etc.

Moreover, under the same storage time, the order of T_2_ values of the mixtures was PS-HKGM < PS-MKGM < PS-LKGM. The larger the Mw of KGM, the smaller the freedom of the water molecules involved in starch retrogradation. KGM with a longer chain might hamper the formation of hydrogen bonds between starch chains due to its steric hindrance, along with combinations with starch chains, giving rise to reduced diffusion and exudation of water in starch paste [34].

### 2.6. Crystallinity

**Figure 5:**
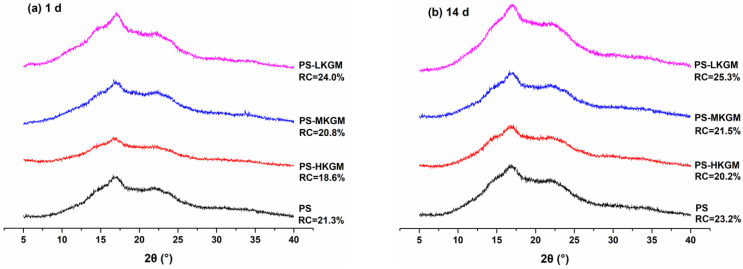
XRD spectra and relative crystallinity of PS-KGM mixtures stored for one day (**a**) and 14 days (**b**). (HKGM, MKGM and LKGM represent KGM with high, medium and low molecular weight, respectively. PS represent pea starch. RC represents relative crystallinity).

X-ray diffraction analysis was performed to determine the type of crystalline structure as well as the degree of crystallinity. The diffractograms of pea starch mixed with different KGM are shown in Figure 5. Native pea starch presents a C-type structure, for both A-type and B-type structures exist in the starch crystal structure, and diffraction peaks appear at the diffraction angles of 5.7°, 15.2°, 17.2° and 23.3° [35]. After gelatinization and regeneration, stronger diffraction peaks at 2θ = 17.0° and weaker diffraction peaks at 2θ = 15.0° and 23.2° appeared. This suggested that the original crystal structure of native pea starch was destroyed and the crystallization type of pea starch transformed from C-type crystallization to B-type crystallization [36,37].

When stored for one day, the mixture of PS-LKGM displayed the highest peak intensity, which indicated the formation of the most crystalline structures. Compared with pure pea starch, the crystallinity increased from 21.3% to 24.0%. After 14 days of storage, the crystallinity of all samples slightly increased with time. This was due to the rearrangement of starch molecules to form an ordered structure during the retrogradation [38]. Moreover, the addition of KGM with medium and high Mw led to the slight decline in pea starch pasta, whereas KGM with low Mw promoted an increase in crystallinity of starch pasta. Due to the various molecular chain lengths of KGM, its spatial conformation and hydrophilicity, interactions between KGM and pea starch inevitably changed, resulting in the discrepancies in pea starch retrogradation behavior.

## 3. Conclusions

The effects of KGM with different Mw on the retrogradation properties of pea starch were explored. At one day of storage, LKGM increased the turbidity, elastic modulus, gel strength and crystallinity of pea starch paste and was accompanied by a decrease in water holding capacity and water freedom, indicating that KGM with low Mw could promote pea starch retrogradation in the short-term. However, during the whole storage period, especially in the late stages, compared with pure pea starch, MKGM and HKGM reduced the turbidity and elastic modulus of pea starch paste and inhibited the increase of gel strength and dehydration. In addition, they also reduced the freedom of water and crystallinity of pea starch. This demonstrated that KGM with medium and high Mw was useful to inhibit the long-term retrogradation of pea starch. The larger the Mw of KGM, the more significant the inhibition effect. In general, the retrogradation of pea starch could be regulated by the different Mw of KGM. This work could provide a reference for the application of polysaccharides like KGM in the processing and storage of starch-based food.

## 4. Materials and Methods

### 4.1. Materials

Commercial Pea Starch (PS) was purchased from Shanghai Yuanye Bio-Technology Co., Ltd. (Shanghai, China). Food-grade konjac glucomannan (KGM) was obtained from Hubei Konson Konjac Gum Co., Ltd. (Ezhou, China).

KGM with different molecular weight (Mw) were obtained according to the method of Chen, with some modification [39]. Native KGM flour with the moisture content of 35% was treated at 121 °C for various durations, including 20 min, 40 min and 80 min. After heterogeneous hygrothermal treatment, konjac flour was dried at 60 °C and sealed. The Mw were determined by Ubbelohde Viscometer to be 1.5 × 10^6^ Da, 1.0 × 10^6^ Da and 3.9 × 10^5^ Da, marked as HKGM, MKGM and LKGM, respectively.

### 4.2. Preparation of PS-KGM Mixtures

The mixtures of PS-KGM were prepared as follows: The above konjac flour was dispersed in distilled water and mechanically stirred to form a sol with a mass fraction of 1%. Afterwards, pea starch, KGM sol and water were blended evenly. Within the mixture, the mass fractions of pea starch and konjac flour were fixed at 5% and 0.5%, respectively [31,33]. After being heated at 100 °C for 30 min, the mixture was dispensed into a beaker with the same volume. Subsequently, the sample was sealed and stored at 4 °C for various durations.

### 4.3. Color Measurement

The lightness (L) value of the sample was measured using a colorimeter (UltraScan XE HunterLab, Reston, VA, USA). Prior to determination, the colorimeter was calibrated by a white plate.

### 4.4. Dynamic Rheological Measurement

Dynamic rheological experiments were performed using a controlled-stress Kinexus 2500 rheometer (Malvern Instruments Ltd., Worcestershire, UK). The experimental parameters were set as follows: the geometry was a cone-plate (CP4/40), the frequency range was 0.1–20 Hz, the strain value was 1% and the temperature was 25 °C. Indicators, such as storage modulus G′ and loss modulus G″, were recorded in real time.

### 4.5. Gel Strength

The gel strength was measured using a TA-XT plus Texture Analyzer (Stable Microsystems Ltd., Surrey, UK). The measurements were performed with a cylindrical probe (P/0.5) at pre-test speed of 1 mm/s, test speed of 1 mm/s, post-test speed of 1 mm/s, compression distance of 4 mm and trigger force of 5 g [40].

### 4.6. Water Holding Capacity (WHC)

The sample was transferred to a centrifuge tube (diameter 30 mm) and subsequently centrifuged at 4800 rpm for 30 min at 25 °C. The supernatant was removed, and the mass of the precipitate was weighed. Water holding capacity (WHC) was calculated as follows:

(1) $$\mathrm{WHC}\;\left(\%\right)=\frac{W_{1}-W_{2}}{W_{1}}\times 100\%$$

where W_1_ represents the weight of sample, and W_2_ represents the weight of supernatant.

### 4.7. Low-Field Nuclear Magnetic Resonance (LF-NMR)

LF-NMR tests were carried out using an instrument (MesoQMR23-060H, Niumag Electric Corporation, Shanghai, China). About 2 g of the sample was packed into a glass tube with a diameter of 15 mm and inserted into the instrument. The tests were operated at 25 °C. The spin-spin relaxation time (T_2_) was recorded using the sequence of Carr–Purcell–Meiboom–Gill (CPMG). Other parameters were set as SW = 100 kHz, SF = 21 MHz, RG = 20 dB, TW = 4000 ms, NS = 4, TE = 0.2 ms and NECH = 18,000.

### 4.8. X-ray Diffractometry (XRD)

XRD patterns of lyophilized samples were measured using a D/Max-IIIA diffractometer (Rigaku, Tokyo, Japan). The tests were conducted under the conditions of 40 kV, 20 mA and the Cu Kα radiation wavelength of 0.1542 nm. The range of the diffraction angle was from 5° to 40° with a step-scan of 0.01°. The relative crystallinity (RC) was calculated using the following equation:

(2) $$\mathrm{RC}\left(\%\right)=\frac{\mathrm{Ac}}{\mathrm{Ac}+\mathrm{Aa}}\times 100\%$$

where Ac represents the crystalline area, and Aa represents the amorphous area.

### 4.9. Statistical Analysis

These data were calculated using an analysis of variance (ANOVA) with the software SPSS 19.0 (SPSS Inc., Chicago, IL, USA) and were presented as the formation of mean plus standard deviations. Statistical differences were defined as *p* < 0.05. Each experiment was carried out at least three times.

## Figures and Tables

**Table 1:** The relaxation time (T_2_) of PS—KGM mixtures at different storage times.

	T_21_ (ms)			T_22_ (ms)			
	PS-HKGM	PS-MKGM	PS-LKGM	PS	PS-HKGM	PS-MKGM	PS-LKGM
1 d	9.22 ± 1.06 ^a^	10.00 ± 2.83 ^a^	12.74 ± 2.46 ^a^	72.39 ± 6.83 ^a^	253.23 ± 10.60 ^a^	335.98 ± 15.55 ^a^	364.25 ± 10.31 ^a^
7 d	6.14 ± 0.92 ^ab^	3.51 ± 0.74 ^bc^	11.75 ± 2.13 ^a^	59.15 ± 4.39 ^a^	200.92 ± 5.94 ^b^	253.23 ± 15.68 ^b^	297.64 ± 6.83 ^b^
14 d	1.75 ± 0.16 ^b^	4.04 ± 0.62 ^b^	5.34 ± 0.11 ^b^	43.29 ± 3.82 ^a^	174.75 ± 10.28 ^bc^	174.75 ± 13.57 ^c^	200.92 ± 5.63 ^c^

T_21_ represents the transverse relaxation time of bound water. T_22_ represents the transverse relaxation time of free water. HKGM, MKGM and LKGM represent KGM with high, medium and low molecular weight, respectively. PS represents pea starch. Values with different letters in the same column were significantly different (*p* < 0.05).

## Data Availability

The data presented in this study are available on request from the corresponding author.
